# Special Issue: Advance in Friction Stir Processed Materials

**DOI:** 10.3390/ma15113742

**Published:** 2022-05-24

**Authors:** Józef Iwaszko, Jerzy Winczek

**Affiliations:** 1 Department of Materials Engineering, Faculty of Production Engineering and Materials Technology, Czestochowa University of Technology, 19 Armii Krajowej Ave., 42-201 Czestochowa, Poland; 2 Department of Technology and Automation, Faculty of Mechanical Engineering and Computer Science, Czestochowa University of Technology, 21 Armii Krajowej Ave., 42-201 Czestochowa, Poland; jerzy.winczek@pcz.pl

## 1. Introduction

In recent years, on the basis of FSP/FSW technologies, a number of new solutions, methods and variants have been developed, constituting not only proof of the continuous evolution of FSP/FSW technologies, but also of the huge scientific and application potential hidden in these methods. The key idea of these works was to induce more beneficial changes in the microstructure of materials and their properties, as well as to ensure better control of the processes taking place at individual stages of machining [1,2]. As is known, the main condition to obtain the assumed material characteristics is appropriate selection of the process parameters and knowledge of the changes in the microstructure of the material and its properties as a function of the machining parameters [3], shape and dimensions of the tool [4,5] or, for example, the sample cooling method [6,7,8,9]. That is why systematic research in this area and experimental verification of developed concepts and ideas are so important. The key objective of this Special Issue was to present the current state of knowledge and the recent advances in developing the microstructure and properties of materials using modern FSP and related technologies. The Special Issue made it possible to collect valuable articles presenting the results of research on changes in the microstructure and properties of the material caused by the application of the above technologies, as well as presenting the cognitive and application potential in addition to underlining the implementation problems and challenges faced by the users of FSP and related technologies.

## 2. Discussion of Research Results

An important factor influencing the degree of grain refinement, i.e., the key parameter characterizing the microstructure formed during FSP, is the method and intensity of sample cooling. The paper [10] presents a solution in which a stream of compressed air, additionally cooled to the temperature of −11 °C with a cooling jet nozzle, was used to cool the 7075 aluminium alloy during FSP. The authors applied two variants of air blowing, i.e., at angles of 45° and 90° to the sample surface and carried out an analysis of the impact of FSP performed under accelerated cooling conditions on the microstructure and properties of the alloy, taking a sample treated with FSP in still air as the reference material. In relation to the material cooled in still air, greater homogeneity of the microstructure of the material and a higher degree of grain refinement were obtained. In the case of the naturally cooled sample, the average grain size in the near-surface zone was 7.6 µm, while in the case of the air-cooled sample it was 1.4 µm in the variant with air blowing at the angle of 45° and 3.2 µm in the variant with air blowing at the angle of 90° with reference to the surface. The consequences of the more favourable changes in the microstructure were higher hardness and resistance to wear resistance of the alloy. The paper also assessed the impact of the blowing angle of the air stream on the microstructure and material properties and indicate that the solution in which the nozzle is inclined at the angle of 45° is particularly favourable because the air stream cools both the material subjected to friction modification and the tool itself. It was shown that such a cooling method leads to stronger grain refinement and better material properties because in such a case there is both direct cooling of the material with the air stream and indirect cooling by means of the tool cooled down by this stream. According to the authors, the proposed cooling method may be a competitive solution compared to other methods because it combines the advantages of compressed air cooling and cryogenic factors.

In turn, Ahmed et al. [11] focused on the use of bobbin tool friction stir welding (BT-FSW) to produce defect-free lap joints in welding AA1050-H14 alloy sheets. The BT-FSW method is an expansion of classic FSW and is increasingly used in industrial practice owing to the number of advantages and benefits of its application. In paper [11], a newly designed and fabricated bobbin tool with different pin geometries (cylindrical, square, and triangular) and concave shoulder profile was used, and then an assessment of the impact of BT-FSW pin geometries on the joint properties was carried out in the context of assessing the possibility of obtaining defect-free lap joints. The authors showed a clear relationship between the temperature at the weld centre, and the pin geometry along with the traverse speed. The authors found, that a square pin leads to a higher BT-FSW stir zone temperature, and the temperature measured at the advancing side is higher than that at the retreating side in all welding conditions. In the case of a tool with cylindrical and square pin geometries, defect-free welds at all the researched traverse welding speeds were obtained.

In paper [12], a friction stir deposition technique (FSD) in the additive manufacturing of AA2011 parts was used. FSD is a solid-state additive manufacturing technique that can be used to deposit different materials. The authors assessed the impact of the feeding speed and the temper condition of the AA2011 alloy on the properties and microstructures of the deposited materials. The conducted research showed that the use of FSD allows the production of sound, continuous multi-layered AA2011 parts without any physical discontinuities or interfacial defects between the layers in all the applied processing parameters. The microstructure of the deposited material was characterized by equiaxed grains, whose size was much smaller in comparison to the grain size measured in the initial material. Despite the intense grain refinement in the case of the AA2011-T6 alloy, lower hardness of the friction stir deposited parts in comparison to the base material was found. An improvement in hardness was obtained only in the case of the AA2011-O friction stir deposited parts and it reached even 163% of the starting material hardness at the applied feeding speed of 1 mm/min.

Analysis of the factors affecting the properties of joints obtained with friction stir lap welding (FSLW) was the subject of research conducted by Choy et al. [13]. An element of a torsion beam shaft of a car chassis produced by joining a pipe made of A357 aluminium alloy with a steel pipe made of FB590 steel of increased strength was tested. The impact of the process parameters on the mechanical parameters of the obtained joint was analysed, as well as on the thickness of the intermetallic compound layer (IMCL) formed in the intermediate layer between the aluminium and steel. The authors placed great emphasis on analysis of the IMCL due to its significant impact on the properties of the joint. The authors found that the thickness of the IMCL, but also the tensile shear load, is primarily impacted by the tool penetration depth. It was found that the thickness of the IMCL decreases with the increase in the tool penetration depth, while the impact of the tool penetration depth on the tensile shear load is more complex. Choy et al. [14] also determined the impact of various process factors on the tensile shear load using definitive screening design. They used four types of tools with different pin lengths. The scope of the experiment adopted by the authors, expressed in its multi-variant nature, allowed a number of relationships to be determined, useful in the control of friction stir lap welding, and facilitating achievement of the assumed goals. The authors discovered, for instance, that the plunge depth has a significant impact on the tensile shear load in addition to the tool penetration depth and also indicated that the plunge depth negatively affects the magnitude of tensile shear load. They also found that the tool penetration depth has the greatest impact on the size of the hooking part in the lap welding of the pipe.

In many industrial applications, aluminium alloys are reinforced with hard ceramic particles to enhance the mechanical properties of aluminium metal matrix composites (Al-MMC). Ali et al. [15] conducted studies on AA6061/SiC/B_4_C composites subjected to friction stir welding. The matrix of the composite was the AA6061 aluminium alloy, while SiC and B_4_C powders were used as reinforcement, whose total share in the composite was 13%. Microstructural investigations were carried out using SEM, as well as radiographic tests, tensile strength tests, and hardness and wear resistance measurements. The microstructural studies revealed strong grain refinement and even particle distribution in the joint area. The use of the reinforcement phase improved the tensile properties of the friction stir welded Al-MMCs. The highest ultimate tensile stress was exhibited by the sample with 10% SiC and 3% B_4_C. It was found that with the increase in B_4_C and decrease in the SiC content in the friction stir welded Al-MMCs samples, the tensile strength declined. The elongation decreased as the percentage of SiC was reduced and B_4_C was raised. The hardness measurements showed growth in its value directly proportional to the B_4_C share and inversely proportional to the SiC content. The coefficient of friction was higher for the samples whose SiC content ranged from 2% to 4%. The conducted research indicated that FSW is an appropriate process for welding Al-MMC, and the obtained results prove the ability to meet the requirements of the aviation and automotive industry. In the opinion of the authors of the work, this technology and the products manufactured thanks to it can also be used in navy and civil navigation owing to the high efficiency of welding.

In [16], an assessment was carried out for the possibility of using the FSW as a welding technique to fill the grooves for welding duplex stainless steel (DSS). For this purpose, three different groove geometries without a root gap were designed and machined in DSS plates with the thickness of 6.5 mm. For comparative purposes, the DSS plate was also welded using the gas–tungsten arc welding (GTAW) method. The obtained joints were subjected to a comparative assessment employing radiographic inspection, optical microscopy, electron back scattering diffraction, as well as hardness and tensile testing. Studies indicated that FSW can be successfully used for welding 2205 duplex stainless steel. It was found that defect-free and sound joints can be produced using a 60° V-shaped groove with a 2 mm root face without a root gap. Compared to the joint obtained by the GTAW method, the FSW joint was characterized by higher yield strength, ultimate tensile strength, and elongation by 21%, 41%, and 66%, respectively. In addition, a clearly higher hardness of the weld joint produced by FSW was found than that produced by GTAW. The maximum hardness of material in the stirring zone in the case of the FSW joint was 280 HV, while in the case of the GTAW joint it was 265 HV.

In papers [17,18], the authors focused on selection of the parameters in friction stir spot-welding (FSSW) processes. Ataya et al. [17] joined a low-carbon steel sheet (A283M-Grade C) with a brass sheet (CuZn40) by means of FSSW using different rotational speeds and dwell times to explore the effective range of parameters, enabling joints to be obtained with a high load-carrying capacity. The quality of the joints was assessed by visual examination, macro- and micro-structural investigations, EDS analysis, the tensile lap shear test, and hardness measurements. Determination of the effect of the total number of revolutions on the heat generated per spot weld was also an important aspect of the work. It was found that the heat input ranged from 11 kJ to 1.5 kJ and it was linearly proportional to the number of revolutions per spot joint. The authors also revealed that when the number of revolutions per spot ranged from 250 to 500, it was possible to produce joints with a high load-carrying capacity from 4 kN to 7.5 kN. In turn, in work [18], FSSW was used to join AA6082-T6 aluminium alloy sheets. Sheets with thicknesses of 1 mm and 2 mm were welded using different rotation speeds, while the dwell time was constant in all the variants. Verification of the effects of FSSW was based on macro- and microstructural investigations, hardness testing and a tensile-shear test. An important element of the characterisation of the process and the manufactured joint was the determination of the heat input generated during FSSW and the peak temperature in the stirring zone. It was found that the highest heat input energy of 3 kJ occurs at the highest rotational speed, and the heat input grows with increasing rotational speed. A similar trend was found in the case of the peak temperature measured in the stirring zone, namely with the increase in the rotation speed, the maximum temperature rose from 236 ± 4 °C to 367 ± 3 °C. The conducted research also showed that the spot joints welded at 600 rpm are characterised by highest the hardness and shear load.

## 3. Summary

Knowledge of the processes, phenomena, and relationships occurring during material processing using FSP/FSW and related technologies is the key to achieving the assumed research and application objectives. Therefore, in this aspect, this Special Issue has become a kind of a platform for exchanging practical experience and knowledge, as well as a source of valuable information that can ultimately become an inspiration to undertake one’s own research in this area. The articles included in this Special Issue indicates that FSP/FSW and related technologies are constantly evolving, and, thus, the possibilities in shaping the microstructure and properties of materials are also growing, which consequently generates new research challenges.
